# Prolonged Survival with Dieting for Improved Autophagy

**DOI:** 10.3390/ncrna11060077

**Published:** 2025-11-04

**Authors:** Akari Fukumoto, Moeka Nakashima, Satoru Matsuda

**Affiliations:** Department of Food Science and Nutrition, Nara Women’s University, Kita-Uoya Nishimachi, Nara 630-8506, Japan

**Keywords:** autophagy, AMPK, ncRNA, miRNA, aging, aging-related diseases, longevity

## Abstract

Food is a crucial component affecting the health of individuals, which may have the potential to expand lifespan. It has been shown that a long lifespan may be related to fine-tuned autophagy. In general, suitable autophagy could play a significant role in the anti-aging biological exertion of the host. AMPK, a member of serine and threonine kinases, could play vital roles within the autophagy signaling pathway in various cells. In addition, alterations in the kinase activity of AMPK have been shown to be connected to several pathologies of aging-related diseases. Therefore, autophagy could control the lifespan-related homeostasis within the host from cells to a body via the modification of AMPK. The design of the diet and/or nutrition targeting the AMPK would be a possibility to expand the lifespan. Some analyses of the molecular biology underlying the autophagy suggest that supplementation of accurate nutraceuticals, as well as dietary restriction, mild fasting, and/or appropriate physical exercise, could modulate AMPK signaling, which may be advantageous for life extension with the alteration of autophagy. Remarkably, it has been revealed that several non-coding RNAs (ncRNAs) might also play significant roles in the regulation of autophagy. In addition, the production of some ncRNAs may be associated with the alteration of gut microbiota with certain diets. Therefore, the modulation of AMPK action with ncRNAs through choosing the relevant diet could be a therapeutic tactic for promoting longevity, which is also accompanied by a reduced risk for several aging-related diseases.

## 1. Introduction

Aging refers to the gradual weakening of physiological integrity and/or functionality, which might, as a consequence, increase susceptibility to various aging-related diseases. Aging is a flexible process that can be determined by genetic, environmental, and/or lifestyle factors [1]. Among them, food nutrition and lifestyle factors may be important components affecting health, which could also increase the lifespan [2]. Interestingly, it has been shown that caloric restriction can significantly extend the lifespan of various species, including humans [3,4]. Additionally, appropriate physical exercises may also improve one’s health markers, which has been suggested to prolong the human lifespan [5]. In terms of their biological mechanisms, it has been discovered that the lifespan may be related to corrected autophagy in cells [6]. Autophagy can actually play a vital role in the anti-aging process by degrading damaged proteins and organelles [7]. Therefore, autophagy has been commonly thought to enhance health and/or lifespan expansion [8]. In addition, an aging-related decrease in autophagy power might be an important initiator of cellular senescence, which may lead to the development of various aging-related diseases [9]. Autophagy is considered a cell survival mechanism that could contribute to the duration of various cells/tissues in organisms [10].

**Figure 1:**
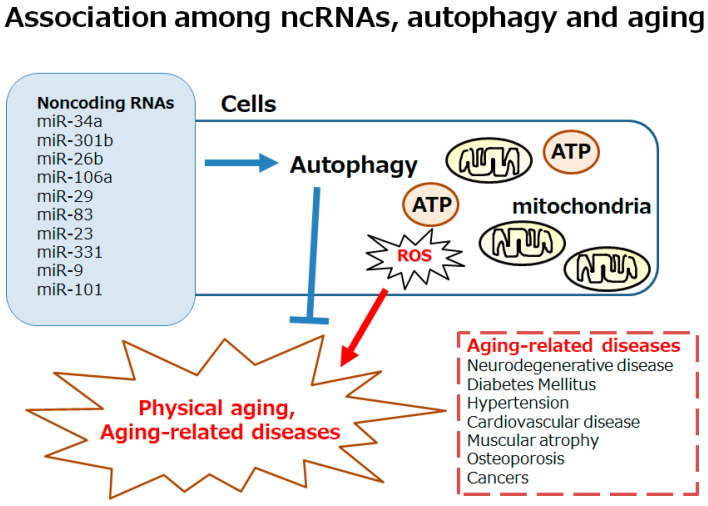
Schematic image of the autophagy involved in both physical aging and aging-related diseases, including neurodegenerative disease, diabetes, hypertension, cardiovascular disease, muscular atrophy, osteoporosis, and cancers. Excess reactive oxygen species (ROS) production may be involved in the development of autophagy. The autophagy could also be affected by various ncRNAs including miR-34a, miR-301b, miR-26b, miR-106a, miR-29, miR-83, miR-23, miR-331, miR-9, miR-101, etc. Note that some important factors including inflammation and/or redox imbalance triggering several aging-related diseases have been omitted for clarity.

Recent studies have revealed the role of non-coding RNAs (ncRNAs) such as microRNAs (miRNAs) in regulating cellular integrity by targeting various components in the autophagy pathway. In addition, the role of ncRNAs in controlling aging processes has also been demonstrated with the discovery of the specific miRNA that regulates lifespan in the nematode *Caenorhabditis elegans* (*C. elegans*) [11]. The degradation of the miRNA has been shown to contribute to aging-related neurodegeneration, affecting the mechanism responsible for the development of various neurodegenerative diseases [12]. For this reason, neurodegeneration could be considered an RNA disorder, whereby the miRNA plays a critical role in the pathology [13]. In the brain, dopaminergic neurons may also be dependent on the function of the relevant miRNA network [14]. Therefore, miRNAs could provide therapeutic targets for neurodegenerative diseases. In general, miRNAs are single-stranded RNA molecules that can regulate gene expression through complementary binding sequences in the three prime untranslated region (3′UTR) of the target genes, potentially modifying the disease phenotype [15]. Furthermore, miRNAs could regulate various biological processes, such as cell proliferation, differentiation, apoptosis, and cell migration, by binding to specific sites of target mRNAs [16]. Interestingly, the silent mating type information regulation 2 homolog-1 (SIRT1) may be a target of miR-34a [17], which could affect cellular physiological senescence and/or aging [18,19]. Up to the present time, several miRNAs, including the SIRT1, have been found to be regulated within the process of aging, which have appeared as key regulators of aging at the cellular and/or tissue levels [20]. A better comprehension of the molecular interaction with the relevant ncRNAs for autophagy and/or aging-related pathologies may be imperative for preventing aging-related diseases, as well as for promoting the extended lifespan of individuals (Figure 1). This review summarizes and discusses the most important nutritional components for improved autophagy, which could also be associated with the longevity of human individuals. In particular, it is reasonable to seek safe and practical nutraceuticals that could induce the appropriate autophagy for improving longevity. This review might also be beneficial for designing a daily diet intended to decrease the rate of aging-related tissue/organ damage.

## 2. Relationship Between Autophagy and Aging-Related Diseases by the Modulation of AMPK Signaling Pathway

Aging may be characterized by the functional weakening of an individual’s cells/tissues/organs, in which aging-related diseases, such as cardiovascular disease, diabetes, neurodegenerative disease, and/or cancer, might frequently develop. Several characteristics of these diseases and/or physical aging are often associated with the alteration of autophagy. In general, autophagy is an evolutionarily conserved host defense mechanism, which may also play important roles for some biological processes. For example, autophagy may involve several cell components being sequestered in auto-phagosomes for molecular recycling [21]. Consequently, the suitable induction of autophagy can extend the individual’s lifespan, whereas its deficiency may reduce the lifespan [22]. Therefore, continued and weakened autophagy could initiate aging-related alterations in cells/tissues, following various disorders in their tissues/organs [23]. In addition, altered autophagy may lead to an increase in abnormal proteins to several organelles, which can eventually exacerbate further aging-related diseases [24,25]. It has been described that a coronary artery disease may be predisposed by the level of autophagy [26]. In addition, appropriate induction of autophagy could contribute to protection against the heart failure of coronary artery disease [27,28]. Autophagy could also protect against neuronal cell damage in the brain of seniors [29]. Levels of autophagy may gradually decrease in mature lymphocytes of seniors. Interestingly, aging-associated memory waning can be decreased by the suitable autophagic effect of spermidine treatment [30]. Consistently, the appropriate autophagy has been reported to be able to improve aging-related dementia in seniors [31]. Furthermore, autophagy may also be involved in the regulation of metabolic diseases such as obesity and/or metabolic syndrome [32]. Autophagy could even protect against aging-related skeletal muscle atrophy [33]. Remarkably, it has been shown that corrected autophagy could be linked to the inverse of immune senescence in humans [34]. Accordingly, autophagy may be essential for the maintenance of physical health with several advantages for the longevity of host [35].

**Figure 2:**
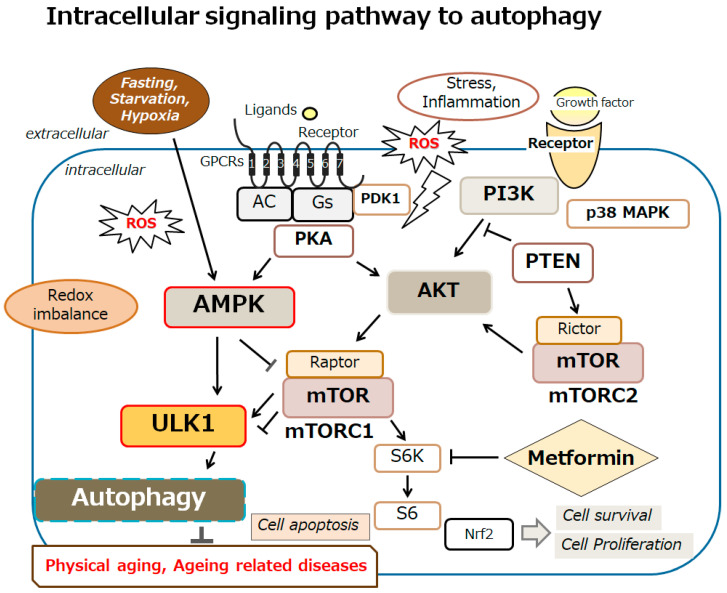
An overview of the intracellular signaling pathway relevant to autophagy. With several key molecules, such as AMPK, PI3K, AKT, mTOR, and ULK1, the autophagy signaling might be involved in the regulation of physical aging and/or aging-related diseases. Inflammation, starvation, fasting, and/or hypoxia are known to initiate the autophagy signaling pathway. Arrowhead indicates stimulation, whereas hammerhead shows inhibition. Note that several signaling pathways such as cytokine-induction and/or inflammatory responses have been omitted for clarity. Abbreviation: mTOR, mammalian/mechanistic target of rapamycin; PI3K, phosphoinositide-3 kinase; AKT, protein kinase B; ROS, reactive oxygen species; AMPK, adenosine monophosphate-activated protein kinase; ULK1, autophagy activating kinase 1; mTORC1, mTOR complex 1.

Adenosine monophosphate-activated protein kinase (AMPK) might be governing in a corresponding intracellular signaling network, sustaining the homeostasis of cells [36]. In particular, AMPK can adjust an energy requirement by approving the ATP production and by regulating the ATP consumption in cells [36,37]. The AMPK can be involved in directing an inhibitory signaling pathway with the mechanistic/mammalian target of rapamycin (mTOR), which may effectively decrease apoptosis by regulating autophagy [38]. Also, AMPK can start the autophagy via the autophagy activating kinase 1 (ULK1) [38,39]. Activation of aserine/threonine kinase ULK1 is an initiation for the formation of phagophores, which are then extended to autophagosomes [40]. The AMPK and mTOR complex 1 (mTORC1) can work as the key mTOR antagonists controlling the ULK1 activity [41]. Via external growth factor stimulation and following the activation of PI3K/AKT and AMPK signaling, mTORC1 could control the phosphorylation and/or the activation of the ULK1 [42] (Figure 2). Therefore, AMPK may be a key regulator in several oxidative stresses for maintaining energy homeostasis, which might be recognized as an energy sensor as a result of its critical roles [43,44]. Remarkably, it is well known that AMPK can provide several protective effects in various cells by governing energy homeostasis. In addition, AMPK can organize several cellular key processes, including protein synthesis, DNA repair, and cell differentiation/proliferation [45,46]. As a result, AMPK could accomplish cell protection, which can respond to many cell damages, including oxidative stress [47]. These vital roles of AMPK in cells/tissues/organs may be indispensable for maintaining the physiological/pathological situation [48,49]. Unquestionably, the regulation of AMPK may play a substantial role for achieving longevity. Physical aging can be renovated by modulating the AMPK signaling pathway, corresponding with the alteration of autophagy [50].

## 3. Several ncRNAs Involved in Longevity via the Modulation of Autophagy

ncRNAs can induce the degradation of mRNAs for the repression of the translation, which could target specific mRNAs through the sequence-specific binding to the 3′ untranslated region (3′UTR) of a mRNA [51]. In general, the biogenesis of ncRNAs together with miRNAs may start with their transcription in the nucleus [52], which is further processed by an RNase enzyme to generate a precursor of the ncRNA [53]. The precursors of ncRNAs are exported to the cytoplasm to generate a mature ncRNA with the help of another RNase enzyme, DICER [54]. Some ncRNAs can bind to its specific target mRNA to form the RNA-induced silencing complex (RISC) [55]. In these ways, some ncRNAs could regulate the mRNAs related to autophagy [56]. Certain ncRNAs have been shown to be upregulated in age-associated diseases, such as Parkinson’s disease [57]. In particular, an increased expression of miR-301b, miR-26b, and miR-106a can regulate autophagy, which might aggravate the alpha-synuclein pathology of Parkinson’s disease [57]. These ncRNAs could also regulate protein folding, which could serve as potential therapeutic targets for protein misfolding in several age-associated diseases. Autophagy may also play an imperative role in the pathogenesis of aging-related macular degeneration that has the harmful aggregation of damaged proteins in retinal cells. In *C. elegans*, secreted ncRNAs such as miR-29 and/or miR-83 could improve the aging-related decrease in autophagy throughout different tissues for longevity [58]. Additionally, loss-of-function mutations in miR-34 can significantly extend the lifespan, which has been shown to bind with the autophagy-related mRNA [59]. Some ncRNAs are also known to play roles in ultraviolet ray A (UVA) and ultraviolet ray B (UVB)-induced photo-related aging in skin fibroblasts by regulating autophagy [60]. In this case, the level of miR-23 might be upregulated during skin exposure to UVA/B irradiation, which is a positive regulator of autophagy in skin [60]. Inhibition of miR-23 could stimulate the activation of autophagy, which might protect the skin fibroblasts from UVA/B-induced cellular senescence [60]. The knockdown of miR-23 is known to increase autophagy in lens epithelial cells, which might alleviate oxidative stresses in the lens [61]. As a result, miR-23 may play a role in the age-associated increase in oxidative stresses in lens epithelial cells, where one of the major causes of eye cataracts with aging might occur. The inhibition of miR-331-30 and miR-9-5p has been shown to prevent the progression of Alzheimer’s disease by activating the autophagy essential for the clearance of amyloid beta proteins [62]. It has been shown that miR-331-3p and miR-9-5p can affect autophagy receptors, which could act as potential markers of Alzheimer’s disease [62]. Another miRNA, miR-101, could also inhibit autophagy. A reduction in miR-101 has been reported in the hippocampus with Alzheimer’s disease [63]. Mimicking the age-associated loss of miR-101 in hippocampal neurons can cause cognitive decline in the model mice of Alzheimer’s disease [63] (Figure 1).

Featuring the role of miRNAs for the regulation of autophagy in various neurodegenerative diseases has been evaluated [64]. It has been shown that several miRNAs are also dysregulated in aging-related diseases with the alteration of autophagy [65]. In addition, some miRNAs can play a dynamic role in cardiovascular disease via the alteration of autophagy [66]. Interestingly, the induction of autophagy by some drugs such as rapamycin could work for longevity through various post-transcriptional mechanisms including the modulation of miRNAs [66,67]. The alliance of miR-506 may be related to the development of atherosclerosis through the regulation of autophagy [68,69]. Furthermore, it has been shown that football training might downregulate the expression of miR-1303, indicating the molecular mechanism of the physical training linking to autophagy and longevity [70]. It has also been shown that a secreted miR-29 and/or miR-83 could influence the aging-related decrease in stresses with the modulation of autophagy in *C. elegans* [58,71]. Up to the present time, various miRNAs have been detected for the regulation of aging, suggesting that some of these may become beneficial indicators and/or regulators for longevity [20,72].

## 4. Possible Tactics with Certain Dieting for Longevity

**Figure 3:**
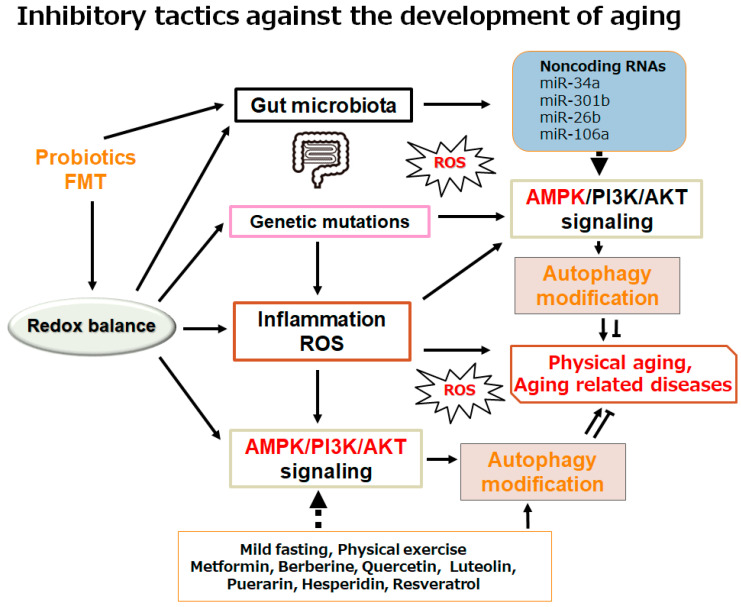
Schematic demonstration of the potential inhibitory tactics against the development of physical aging and/or aging-related diseases. Example implements including metformin treatment and several nutraceuticals dieting as well as mild fasting/physical exercise known to act on the autophagy modification are also shown. Note that some significant activities such as inflammatory responses and/or reactive oxygen species (ROS) production from inflammatory conditions have been misplaced for clarity. Some probiotics and/or fecal microbiota transplantation (FMT) could support the positive modification of gut microbiota for the improved autophagy via the alteration of non-coding RNA production, which might also be advantageous for the treatment of physical aging and/or several aging-related diseases.

Agreeing with the notable role of autophagy in the pathogenesis of aging and aging-related diseases, autophagy could work as an inspiring therapeutic target for longevity (Figure 3). In fact, several inhibitors such as metformin can target the AMPK pathway, thereby mediating the modulation of autophagy for the treatment of several aging-related diseases. Remarkably, it has been shown that the modulation of autophagy might be useful both in improving cardiac function and in remedying cardiovascular disease [73]. For example, a study in an animal model of an aging heart has shown that mild fasting with the appropriate induction of autophagy may enhance the cardiac function and/or the durability of the heart, which may be linked to the clearance of damaged cellular components in cardiac cells by autophagy [73]. Here, mild fasting may include short-term periodic fasting and/or intermittent fasting. Hence, the promotion of autophagy by mild fasting could actually lead to longevity.

A popular form of repeated mild fasting has been confirmed to suggest multiple health benefits, including an extension of healthy lifespan, in which several miRNAs may be expressed to work in preclinical models [74]. Remarkably, mild fasting is also effective in the treatment of various solid tumors in mice models [75]. Several cancers could be treated for the prevention of cell proliferation by increasing cellular apoptosis via the autophagy modulatory mechanism with several miRNAs [76]. In addition, mild fasting could activate AMPK signaling for the modulation of autophagy in cancers [74,76], which can respond to various cell damages including oxidative stresses [47]. The ghrelin release can control the mTOR signaling pathway, which could subsequently control the autophagy in chronic liver diseases [77]. The ghrelin could also stimulate glucose metabolism for energy production in the brain, thereby probably preventing the deterioration of memory function in dementia [78]. Interestingly, it has been suggested that the ghrelin can attenuate the TGFβ-induced fibrosis via the miR-125a-5p action by antagonizing the TGF receptor signaling pathway [79].

With regard to the AMPK signaling for the modulation of autophagy, the nutraceutical berberine, for example, a compound observed in a diverse range of herbs used in traditional Chinese medicine, has shown beneficial activity for the regulation of hyperlipidemia and type 2 diabetes via the activation of the AMPK signaling pathway [80]. Actually, the berberine could stimulate AMPK signaling in a manner equivalent to metformin [81]. The capability of berberine to promote autophagy has been recognized in rodent animal models in vivo [82]. The berberine could protect several tissues from oxidative injury by inducing autophagy. In addition, it has been shown that the berberine may possess a therapeutic effect in cardiovascular disease [83]. The berberine may also serve the cardioprotective effect for the myocardial damage from an ischemia/reperfusion of the heart by inducing autophagy [84,85]. The miR-29b expression can be increased by the berberine treatment, which may be abolished by the usage of AMPK inhibitors [86]. Flavonoids are broadly present in various grains, vegetables, fruits, and medicinal plants, which may have the potential to work as protective mediators against several aging-related diseases. In particular, specific flavonoids, such as quercetin, puerarin, luteolin, and hesperidin, have verified the cardioprotective activity in animal models [87]. In addition, quercetin can induce neurogenesis, enhancing the longevity of neuronal cells, probably by modulating the AMPK signaling pathway [88]. The antioxidative nutraceutical components in *Humulus japonicus*, such as luteolin, have been shown to be able to scavenge reactive oxygen species (ROS) within various cellular components, including mitochondria [89]. In neurodegenerative diseases such as Alzheimer’s disease, it has been shown that these flavonoids have the potential to modify the regulation of several miRNAs [90]. However, there are considerable alterations in the expression of miRNAs during antioxidant responses triggered by flavonoids [14,91]. Neuronal cells have been shown to exhibit 14 miRNAs linked to the antioxidant system with flavonoid treatment when oxidative stresses occur [92]. It has been suggested that miRNA-dependent anti-inflammatory mechanisms can confirm the neuroprotective effects of flavonoids [93]. Therefore, these modifications of miRNAs may indicate that certain flavonoid treatments can induce the differential expression of miRNAs that can contribute to the development of/decrease in several neurodegenerative diseases [94]. In the same way, luteolin can improve the lipid accumulation in nonalcoholic fatty liver disease (NAFLD) by increasing the oxidation of fatty acids via the increased mitochondrial biogenesis by upregulating the AMPK signaling pathway [95]. Furthermore, puerarin has been shown to be a promising flavonoid for increasing the longevity of *Drosophila melanogaster* by stimulating autophagy [96]. Interestingly, puerarin can decrease the level of miR-7, which may activate AMPK signaling for the regulation of autophagy [97]. It has been shown that hesperidin could also slow down the aging speed with prolonged longevity via the modulation of AMPK signaling [98,99]. Damaged lung tissue in mice may show a considerable increase in several miRNA levels within the injury place, suggesting that the modulation of miRNAs for enhanced autophagy has a protective effect with the use of hesperidin [100]. Similarly, it has been described that resveratrol may also have a protective effect on cardiac dysfunction by modifying autophagy [101]. Resveratrol could protect mitochondria by modulating autophagy through the alteration of AMPK signaling in myocardial cells [102]. Additionally, resveratrol can inhibit hyperglycemia-induced cardiomyocyte hypertrophy by reducing oxidative stresses via the stimulation of autophagy to maintain the mitochondrial homeostasis [103]. Resveratrol could also improve retinal arterioles, which can eventually protect against aging-related retinal neurodegeneration via the alteration of the AMPK-dependent signaling pathway [104]. As a result, certain nutraceutical treatments may indeed have potential for the regulation of aging-related diseases as well as for longevity via the regulation of autophagy (Figure 3).

## 5. Future Perspectives

Accumulating data suggest that regulatory ncRNAs, including various miRNAs, circRNAs, and lncRNAs, can affect the host–microbiota as well as microbiota-associated diseases, including cancers and/or diabetes [105]. Interestingly, the expression of several ncRNAs could be repressed by certain gut microbiota in the host [106]. It has also been shown that there are intricate connections between the gut microbiota and the expression of ncRNAs in a host, suggesting that gut microbiota may have a potential role for the defense against aging-related diseases [107]. The elaborate collaboration could even regulate the growth of cancer cells [108] (Figure 3). For example, a study has demonstrated that *miR-30a-3p* expression exerts tumor-suppressive functions in some forms of cancers [109]. In addition, the ectopic expression of *miR-30a-3p* could also attenuate the aggressiveness of several cancer cells [109]. Interestingly, *miR-30a-3p* could enhance the chemosensitivity of cancer cells [110]. Actually, *miR-30a-3p* expression can repress cell growth, migration, and/or inflammatory responses in fibroblastic cells via the alteration of autophagy [110]. Interestingly, nutrient starvation may induce a considerable intensification of autophagy via Ulk1 dephosphorylation with the decreased expression of the miR-30a-3p [111]. In contrast, the upregulation of miR-30a-3p could reverse the effects of autophagy with the use of rapamycin [112]. Rapamycin can promote autophagy, which has, therefore, been used as a potent inducer of autophagy [112]. Thus, some ncRNAs could regulate autophagy. It has been shown that downregulated *miR-30a-3p* expression could promote autophagy in mice mammary glands [113]. On the contrary, autophagy may be inhibited by the overexpression of *miR-30a-3p* in mammary glands [113]. Interestingly, the increased expression of miR-34a or the inhibited autophagy may be associated with aging and/or a metabolic syndrome [114]. The miR34a could repress the expression of the *nicotinamide phosphoribosyl transferase* gene, which may lead to reduced levels of SIRT1 activity [115]. It has been shown that inflammatory transcription factors such as nuclear factor-kappa B (NF-κB) may be involved in the expression of *miR-34a* [116]. The upregulation of *miR-34a* could also increase the activity of NF-κB [117]. Therefore, the overexpression of the *miR-34a* can promote the apoptosis in various cancer cells [118]. Instead, the inhibition of miR-34a expression can not only increase the expression of SIRT1 to induce the suitable activity of autophagy but also strengthen the ability of cells to oppose apoptosis. Consequently, the downregulation of miR-34a could further improve the outcomes of several therapies against the progression of aging-related diseases including cancers. Doubtlessly, cancer prevention might contribute to longevity.

Aging may be a noteworthy promoter for the development of aging-related diseases, whereas aging-related diseases could also exacerbate the aging process further. An increase in the incidence of aging-related diseases may lead to a particular decrease in the quality of life (QOL) of individuals. Therefore, better health care for longevity may ensure a significant elevation in QOL. Aging molecular mechanisms including the control of autophagy at the cellular, tissue, and/or body levels should be accurately characterized in order to develop novel strategies for longevity. Although it should be noted that some beneficial effects might be related to the modification of the cellular antioxidant system, particular nutraceutical regimens within physiological doses may have significant potential for the treatment of aging-related diseases. Some natural bioactive molecules could induce autophagy, which may also improve several aging-related diseases via the alteration of gut microbiota. Again, a longevity-promoting effect with appropriate autophagy could be provided, in which ncRNAs might play imperative roles. Nonetheless, the relationship among autophagy, ncRNAs and gut microbiota should be intensely investigated in future studies.

## 6. Conclusions

The conclusion of this review is that supplementation of certain nutraceuticals and/or mild caloric restriction could modify the autophagy for longevity. In other words, prolonged survival could be achieved by certain dieting with improved autophagy. Several ncRNAs might play imperative roles in the process of autophagy regulation. Specific dieting could also be associated with the alteration of gut microbiota, which may provide beneficial ncRNAs for the reduced risk of several aging-related diseases via the alteration of autophagy in relevant tissue cells.

## Data Availability

No new data were created or analyzed in this study. Data sharing is not applicable to this article.

## Abbreviations

AMP	adenosine monophosphate
ATP	adenosine triphosphate
AMPK	adenosine monophosphate-activated protein kinase
*C. elegans*	*Caenorhabditis elegans*
FMT	fecal microbiota transplantation
miRNA	microRNA
mTOR	mechanistic/mammalian target of rapamycin
mTORC1	mTOR complex 1
NAFLD	nonalcoholic fatty liver disease
QOL	quality of life
ROS	reactive oxygen species
3′UTR	three prime untranslated region
ULK1	autophagy activating kinase 1
UVA	ultraviolet ray A
UVB	ultraviolet ray B
